# 

**Published:** 1964-01

**Authors:** 


### Contents

| | | |
|---|---|---|
| CAVERS DYING OF COLD . . . . . . | *O. C. Lloyd* | 1 |
| ADRENOCORTICAL HYPERFUNCTION AND OAT-CELL CARCINOMA OF THE BRONCHUS . | *D. Mattingly et al.* | 6 |
| TWENTY YEAR FOLLOW-UP OF A CASE OF MASSIVE CALCULUS IN A VESICAL DIVERTICULUM . . . . . . . | *A. W. Adams* | 15 |
| CHLORPROPAMIDE IN PARKINSON'S DISEASE | *P. C. Barrington* | 16 |
| REPORTS FROM SOCIETIES . . . . . . . | | 20 |
| BARROW HOSPITAL . . . . . . . . | | 21 |
| SOUTHMEAD HOSPITAL . . . . . . . | | 22 |
| NOTES AND NEWS . . . . . . . . | | 23 |
| APPOINTMENTS . . . . . . . . . | | 23 |

